# Environmental surveillance reveals enterovirus diversity in Jinan, China: detection of types D68, A71, A76, B88, A90, and C99

**DOI:** 10.1128/spectrum.01928-25

**Published:** 2025-12-18

**Authors:** Mengmeng Wang, Feng Ji, Meng Chen, Yao Liu, Xiaojuan Lin, Haifeng Hou, Zexin Tao

**Affiliations:** 1 School of Public Health, Shandong First Medical University and Shandong Academy of Medical Sciences 373247, Jinan, China; 2 Shandong Center for Disease Control and Prevention 518873, Jinan, China; Fujian Agriculture and Forestry University, Fuzhou City, China

**Keywords:** enterovirus, environmental surveillance, next-generation sequencing, genotype, phylogeny

## Abstract

Enterovirus (EV) exhibits high evolutionary activity and is associated with a wide spectrum of human diseases. Environmental surveillance employing next-generation sequencing (NGS) is an effective approach for elucidating EV type diversity and genetic variation; however, similar studies in China are limited. Sewage samples were collected monthly in Jinan, China, from January to December 2024 and subsequently concentrated. EV isolation was conducted using cell culture, followed by VP1 amplification and Sanger sequencing. Concurrently, VP1 amplicons directly obtained from sewage concentrates were subjected to NGS. Similarity and phylogenetic analysis were performed. A total of 104 EV strains representing nine serotypes were isolated via cell culture, with echovirus 11 (E11) and E3 being the most prevalent, accounting for 34.62% (36/104) and 27.88% (29/104) of the total isolates, respectively. NGS technology identified 31 EV types, with CVB4 and CVA16 exhibiting the highest read counts. Additionally, uncommon types such as D68, A71, A76, B88, A90, and C99 and 11 rhinovirus types were detected in the sewage samples. Phylogenetic analysis revealed that CVA16 and D68 sequences from sewage had close genetic relationship with recent Chinese strains, and multiple branches of B88 and C99 were observed. This study represents the first detection of D68 and B88 in sewage in China, demonstrating co-circulation of diverse enteroviruses with significant genetic variability in the region. These findings underscore the utility of environmental surveillance for tracking EV epidemiology and evolution.

**IMPORTANCE:**

Enteroviruses are significant human pathogens associated with frequent epidemics and outbreaks. Although numerous types exist, some exhibit low prevalence and are rarely detected. By implementing next-generation sequencing (NGS) in wastewater surveillance, this study comprehensively characterized enterovirus diversity, revealing multiple uncommon types of interest. Notably, we detected EV-D68—an emerging agent linked to acute flaccid myelitis—in sewage, underscoring its public health relevance. Additionally, we identified multiple rhinovirus types. These data demonstrate the local circulation of diverse viruses with substantial public health implications and provide a critical foundation for disease monitoring and early warning systems.

## INTRODUCTION

Enteroviruses (EVs) can cause a variety of important human diseases, including poliomyelitis, aseptic meningitis, and hand-foot-and-mouth disease (1). EVs are non-enveloped, single-stranded positive-sense RNA viruses with an icosahedral capsid structure and a particle diameter of 27–30 nm. They are classified within the genus *Enterovirus* under the order *Picornavirales*. The complete genome of EV is approximately 7,500 bp in length and contains a long open reading frame spanning ~6,500 bp, flanked by 5′ and 3′ untranslated regions (5′-UTR and 3′-UTR) (1). The structural protein-coding region, P1, encodes four capsid proteins VP4, VP2, VP3, and VP1. Among these, VP1 is the most externally located protein and harbors the principal antigenic determinants, making it a key target for genotyping and molecular epidemiological studies. In 1999, Oberste et al. introduced a molecular typing method for EVs based on VP1 sequence similarity, defining strains of the same type as sharing >75% nucleotide identity, whereas those of different types exhibit <70% similarity (2).

Individuals infected with EVs can shed large quantities of viral particles in their feces for weeks, facilitating viral entry into urban sewage systems. Given this mode of transmission, environmental surveillance of sewage serves as a valuable tool for monitoring EV prevalence in populations. Recognizing its importance, the World Health Organization (WHO) recommended in 2003 that environmental surveillance be implemented as a complementary strategy to acute flaccid paralysis (AFP) case monitoring. Many countries have implemented sewage surveillance, achieving successful applications in monitoring the circulation of wild poliovirus (PV) and vaccine-derived poliovirus (VDPV) (3). China initiated environmental surveillance of poliovirus and other enteroviruses in 2008, with Shandong and Guangdong provinces pioneering these efforts and reporting successful outcomes (4, 5).

Currently, the cell culture-virus isolation method remains the most widely used technique for EV environmental surveillance worldwide. However, this approach has notable limitations. It exhibits a preferential detection of species *EV-B* due to their more pronounced cytopathic effect (CPE) and is susceptible to inter-serotype growth competition. Consequently, cell culture can skew surveillance results by overlooking non-*EV-B* serotypes (6). It is precisely this limitation that underscores the value of next-generation sequencing (NGS) as a powerful alternative. By directly extracting and sequencing EV nucleic acids from concentrated sewage samples, NGS bypasses the cultivation biases inherent to cell culture. This thereby enables a more comprehensive and unbiased assessment, allowing for the detection of a broader range of EV types, as demonstrated in our subsequent results (7, 8).

Throughout the three-year COVID-19 pandemic, the prevalence of EVs in the general population exhibited a marked decline, whereas the incidence of EV-associated diseases remained consistently low (9, 10). Our previous surveillance data corroborated this trend, demonstrating reduced EV detection in wastewater samples during this period (11). Following the relaxation of non-pharmaceutical interventions (NPIs) in China since 2023, we have observed a resurgence of multiple infectious diseases, including EV infections (11–13). This epidemiological shift underscores the critical importance of investigating EV serotype diversity and phylogenetic characteristics through urban wastewater surveillance.

In this study, we conducted monthly collection of urban wastewater samples throughout 2024 in Jinan, the capital of Shandong Province. The viral content was concentrated from these samples, followed by parallel examination using both cell culture techniques and NGS approaches. This dual-method strategy enables comprehensive monitoring of circulating EV strains while simultaneously enriching China’s molecular epidemiological database for EVs.

## MATERIALS AND METHODS

### Sewage collection and concentration

In this study, the No. 2 Everbright Sewage Treatment Plant in Jinan, which serves a population of approximately one million, was selected as the sampling site, where influent samples were monthly collected using the grab method, with 1 L of raw sewage collected in sterile sampling bags and transported to the laboratory under cold conditions (4°C) for immediate processing.

Sewage concentration was performed using mixed cellulose ester (MCE) membrane adsorption and the ultrasonic elution method (6). Briefly, the collected sewage samples were centrifuged at 3,000 × *g* for 30 min at 4°C to pellet suspended solids, after which the supernatant was transferred to a sterile beaker, and MgCl₂ and hydrochloric acid solution were slowly added to adjust the pH to 3.5. Qualitative filter paper, MCE membrane, and pre-filter membrane were sequentially installed into the filter device. The sewage supernatant with adjusted pH was transferred to the filter device and filtered to adsorb viruses onto the anion exchange membrane. After filtration, the middle anion exchange membrane was removed and cut into pieces using sterile scissors. The pieces were added to 10 mL of 3% beef extract solution with a pH of 8.5. The viruses adsorbed to the filter membrane were eluted by sonicating for 3 min on ice using an ultrasonic disruptor. The eluate was centrifuged at 3,000 × *g* for 30 min at 4°C. The supernatant was filtered through a 0.22 µm disposable syringe filter to remove bacteria. Approximately 100–200 μL of 0.5 M hydrochloric acid solution was added to adjust the pH to 7.0. The eluate was then transferred to a 4.8 mL NUNC cryovial and stored at −80°C.

### Virus isolation, entire VP1 RT-PCR, Sanger sequencing, and typing

Human rhabdomyosarcoma (RD) cells, human laryngeal carcinoma epithelial cells (HEp-2), and mouse lung cell line expressing human poliovirus receptor (L20B) were used for EV isolation. The sewage concentrate was inoculated into RD and HEp-2 cell culture plates at 200 µL × 12 vials each and incubated at 37°C with 5% CO₂. The cells were examined daily under a microscope for CPE. After 5 days, cultures were frozen and thawed and repassaged onto the same cell lines for another 5-day period. Cultures with CPE were passaged onto L20B cells to determine whether they were PV or non-polio enterovirus (NPEV). Viral RNA was extracted from 200 µL of cell culture supernatant using an automated nucleic acid extraction system (Xian Tianlong, NP968-C) and the Viral DNA/RNA Extraction Kit (v4.0), following the manufacturer’s protocol. Approximately 100 µL of RNA was obtained and stored at −80°C until further processing.

RT-PCR amplification of the complete VP1 coding region was conducted using the SuperScript III One-Step RT-PCR System with Platinum *Taq* DNA Polymerase (Invitrogen, USA). The primer pair UG1/UC11 (14) (forward primer UG1, 5′-TTG GTC AGC GTA ATG A-3′; reverse primer UC11, 5′-AAG AGG TTC TAT TCC ACA T-3′) was used for PV. For NPEV, primer pairs 486/488 and 040/011 were used for *EV-A* types, and primer pairs 008/013 and 197/011 were used for *EV-B* types (2, 15). The reaction conditions were 50°C for 30 min and 94°C for 2 min, followed by 40 cycles of denaturation at 94°C for 15 s, annealing at 50°C for 30 s, and extension at 68°C for 90 s, with a final extension at 68°C for 5 min. The PCR products were analyzed via 1.5% agarose gel electrophoresis, and the positively identified products were forwarded to BGI Genomics for Sanger sequencing. Sequencing results were verified and assembled, with type identification conducted using online BLAST.

### RNA extraction, partial VP1 seminested RT-PCR, and NGS sequencing

Nucleic acids were extracted directly from sewage concentrates using the KingFisher Flex nucleic acid extractor and the MagMAX Pathogen RNA/DNA Kit (ABI, USA). A volume of 90 µL of nucleic acids was extracted from 300 µL of concentrate.

A semi-nested RT-PCR method was utilized to amplify the partial VP1 coding region of EV from the sewage RNA. The primers AN32, AN33, AN34, and AN35, along with SuperScript III reverse transcriptase (Invitrogen, USA), were used for cDNA synthesis (16). The initial and second rounds of PCR amplification were performed using Platinum *Taq* DNA Polymerase (Invitrogen) with primer pairs 224/222 and AN89/AN88, respectively, yielding a final product of approximately 370 bp in length.

The PCR products were subjected to 1.5% agarose gel electrophoresis. Positive products were sent to BGI Genomics for NGS on the DNBSEQ sequencing platform. After sequencing, clean reads were obtained by removing reads containing adapters, reads containing poly-N, and low-quality reads (Q30 >85%). The clean reads obtained were subjected to *de novo* assembly using CLC Genomics Workbench 24.0 (QIAGEN, Germany). The assembled contigs with a length greater than 200 bp and a sequencing depth >30 were identified using a local BLAST database containing the VP1 coding region sequences of all EV types. Additionally, the clean reads for CVA16, EV-D68, EV-B88, and EV-C99 were subjected to resequencing analysis to obtain their consensus sequences.

### Phylogenetic analysis

Similarity and phylogenetic analysis were performed for the four types of CVA16, EV-B88, EV-C99, and EV-D68. The sequences obtained in this study were aligned and compared with sequences obtained in GenBank using Bioedit 7.7.1.0 software. The phylogenetic tree based on the partial VP1 coding region was constructed using the maximum likelihood method in Mega 7.0 software (17). The evolutionary distances were calculated using the Kimura 2-parameter model, and the Bootstrap value was set to 1,000 replicates.

## RESULTS

### EV isolation from sewage

In 2024, a total of 12 sewage samples were collected in Jinan City, all of which tested positive for EV isolation. A total of 104 EV strains were isolated, belonging to nine serotypes. Specifically, two strains of coxsackievirus A4 (CVA4) were classified under species *EV-A* (1.92%), whereas 18 strains of PV1 and 14 strains of PV3 were categorized under species *EV-C* (30.77%). The remaining 70 strains belong to six serotypes of species *EV-B* (67.31%) (Table 1). All 32 PV strains were Sabin-like, with no detection of PV2 or VDPV. In comparison to the Sabin vaccine strains, nucleotide variations in the VP1 region of PV1 ranged from 1 to 3, with the majority of PV1 strains (72.2%, 13/18) exhibiting only a single variation site. The VP1 region of PV3 displayed variations ranging from 0 to 4, with 71.43% (10/14) of PV3 strains having one or fewer variation sites (Table 2). Within the species *EV-B*, echovirus 11 (E11) was the most frequently isolated serotype, accounting for 34.62% (36/104) of the isolates, followed by E3, which constituted 27.88% (29/104). Additional isolates included two strains of CVB4 (1.92%), one strain of CVB5 (0.96%), one strain of E7 (0.96%), and one strain of E18 (0.96%).

**TABLE 1:** Numbers of enterovirus isolates from sewage, by type and by month

Serotype	Jan	Feb	Mar	Apr	May	June	July	Aug	Sep	Oct	Nov	Dec	Sum
CVA4												2	2
CVB4					1		1						2
CVB5		1											1
E11				2			1	3	13	15	1	1	36
E18								1					1
E3		3		1	3		1	13	3	1	4		29
E7							1						1
PV1	1	6		2	2		1	3	2		1		18
PV3	3	1	1	2	1	1	2		1			2	14
Sum	4	11	1	7	7	1	7	20	19	16	6	5	104

**TABLE 2:** Number of nucleotide variations in VP1 sequences of poliovirus in sewage

Serotype	Number of VP1 nt variations					Sum
	0	1	2	3	4	
PV3	5	5	1	0	3	14
PV1	0	13	2	3	0	18
Sum	5	18	3	3	3	32

A distinct seasonal peak in EV isolation was observed during the summer and autumn, with the highest isolation rate occurring in August. During this period, serotype E3 predominated. Subsequently, in September, isolations of E11 increased sharply, ultimately becoming the dominant serotype for the year (Table 1).

### NGS sequencing on partial VP1 amplicons

Nucleic acids were extracted from the concentrates of the 12 sewage samples and subjected to semi-nested RT-PCR targeting a partial VP1 coding region. Electrophoresis confirmed successful amplification in all 12 samples. The amplified products were subsequently sequenced using NGS.

The clean data read counts ranged from 4,246,624 to 12,233,654 per sample, with EV reads varying between 10,020 and 9,715,781. Summarizing all samples, the total read count was 128,602,321, of which 38,945,757 (30.28%) were EV-specific. Additionally, rhinovirus (RV) accounted for 1,186,603 reads (0.92% of the total) (Table 3).

**TABLE 3:** Total number of NGS reads and those belonging to enterovirus and rhinovirus

Months	Total reads	EV reads	%	RV reads	%
JN2401	6,597,168	18,954	0.29	1,148,924	17.42
JN2402	12,067,551	522,921	4.33	52	0.0004
JN2403	12,055,661	4,087,000	33.90	2,779	0.023
JN2404	11,162,616	4,152,507	37.20	\^*a*^	\
JN2405	10,733,289	4,256,777	39.66	17,819	0.17
JN2406	4,246,624	145,703	3.43	\	\
JN2407	10,940,241	3,402,764	31.10	16,785	0.15
JN2408	12,233,654	2,981,352	24.37	\	\
JN2409	12,163,961	8,894,557	73.12	\	\
JN2410	12,089,520	5,236,773	43.32	244	0.002
JN2411	12,224,106	2,616,291	21.40	\	\
JN2412	12,087,930	2,630,158	21.76	\	\
Sum	128,602,321	38,945,757	30.28	1,186,603	0.92

^*a*^ \, no sequencing reads were detected.

**Fig 1:**
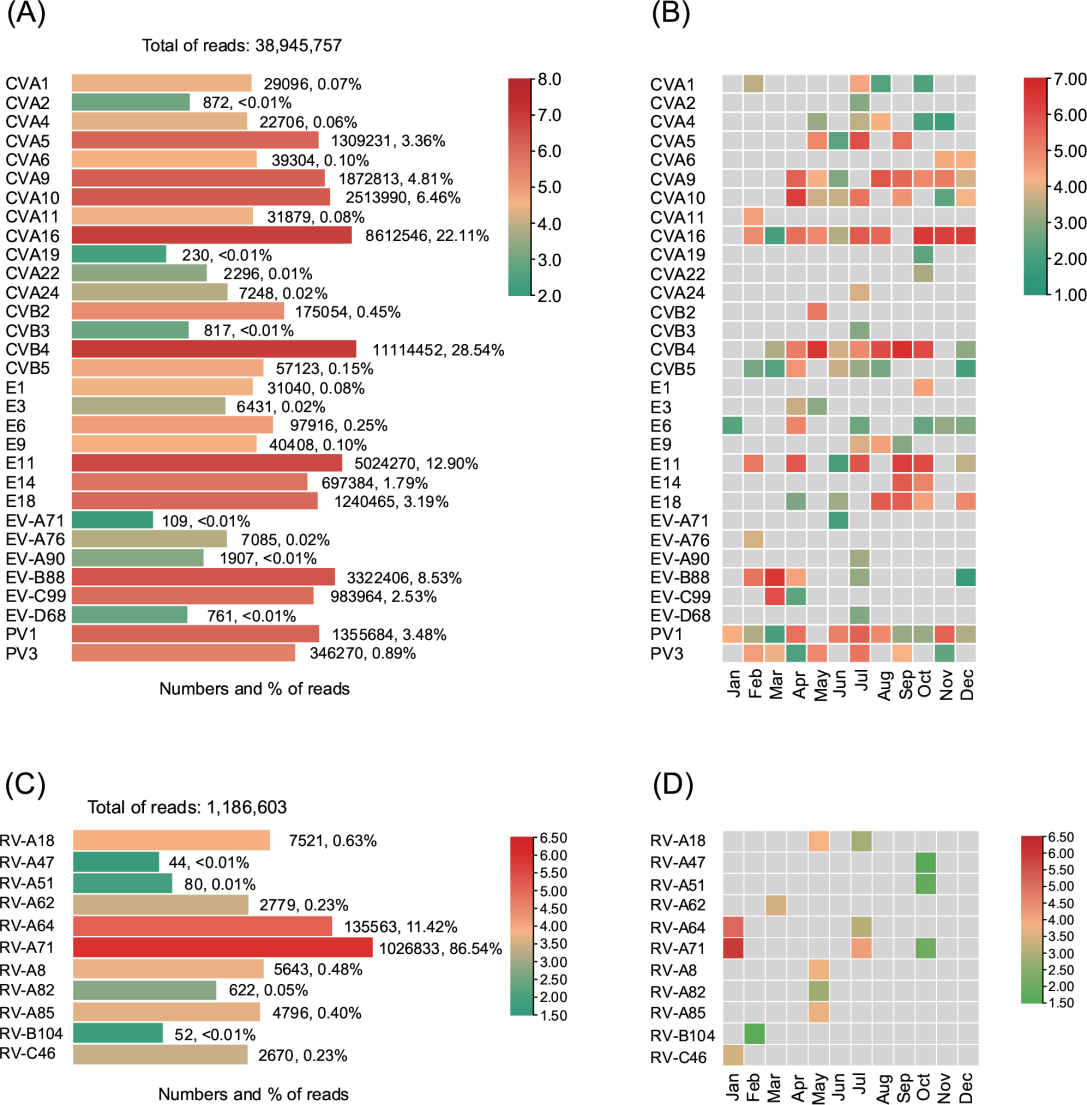
Enterovirus types detected from sewage samples. Total number and constituent ratio of reads mapped to different enterovirus and rhinovirus types are shown in the left panels of (**A **and **C**), respectively. Panels (**B**) and (**D**) illustrate the results of the logarithm of the number of NGS reads belonging to different types of enterovirus and rhinovirus types in every month during 2024, respectively.

BLAST analysis identified 31 EV types and 11 RV types from the sewage RNA amplicons. The detected EV types spanned four species: *EV-A* (including CVA2, CVA4-6, CVA10, CVA16, EV-A71, EV-A76, and EV-A90), *EV-B* (including CVA9, CVB2-5, E1, E3, E6, E9, E11, E14, E18, and EV-B88), *EV-C* (including CVA1, CVA11, CVA19, CVA22, CVA24, PV1, PV3, and EV-C99), and *EV-D* (EV-D68). Compared with cell culture, NGS failed to detect E7 but identified all other EV serotypes. Among EVs, CVB4 had the highest read count (11,114,452; 28.54% of total EV reads), followed by CVA16 (22.11%) and E11 (12.90%) (Fig. 1A). The temporal distribution revealed that PV1, CVA16, and CVB4 were the most prevalent serotypes, detected in 11/12, 10/12, and 9/12 samples, respectively. Among the newer EVs, EV-B88 demonstrated a relatively high detection frequency, being identified across 5 different months (Fig. 1B).

The RV types included RV-A18, RV-A47, RV-A51, RV-A62, RV-A64, RV-A71, RV-A8, RV-A82, RV-A85, RV-B104, and RV-C46, with RV-A71 dominating at 86.54% of RV reads (1,026,833/1,186,603) (Fig. 1C). Compared with EV detection, RV exhibited lower detection frequency, with individual serotypes detected only 1–3 times (Fig. 1D).

### Similarity and phylogenetic analysis

We performed similarity and phylogenetic analyses based on partial VP1 nucleotide sequences of this study, together with those from the GenBank database. These analyses focused on both predominant serotypes (CVA16) and uncommon types (EV-B88, EV-C99, and EV-D68).

**Fig 2:**
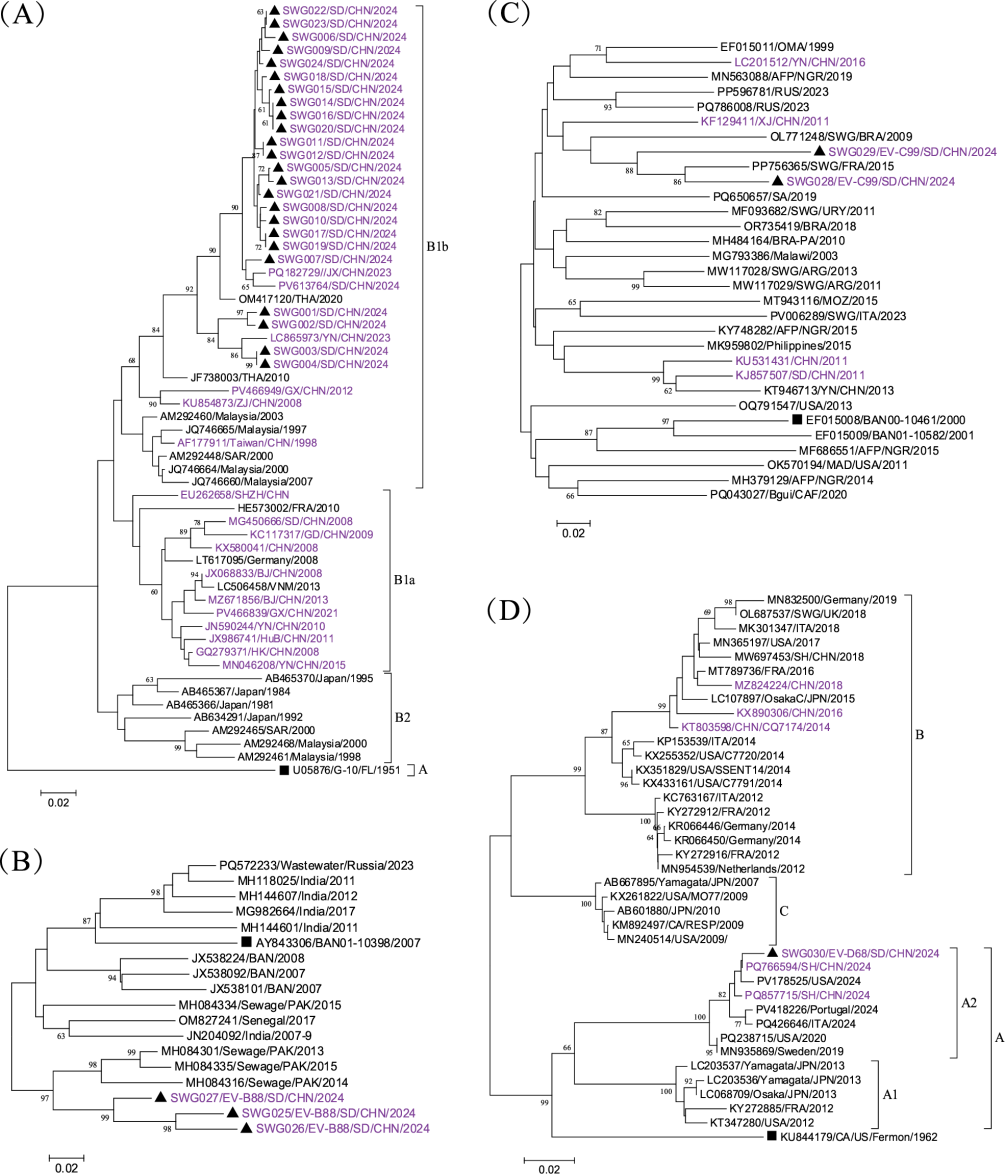
Phylogenetic analysis based on partial VP1 coding region of CVA16 (**A**), EV-B88 (**B**), EV-C99 (**C**), and EV-D68 (**D**). Squares represent prototype strains, triangles indicate the sequences in this study, and taxon names in purple indicate sequences in China.

Our sequence analysis included 24 clinical CVA16 strains (315 bp partial VP1 region, corresponding to nt 2593–2907 of prototype strain U05876/G-10/FL/1951) along with 35 reference sequences from GenBank. The nucleotide similarity among the sewage isolates ranged from 91.4% to 100%, whereas their similarity to other reference sequences varied from 74.6% to 99.3%. Phylogenetic reconstruction demonstrated clear genotypic segregation (Fig. 2A). The prototype strain U05876/G-10/FL/1951 occupied a distinct position as the sole representative of genotype A. The majority of circulating strains, including all sewage isolates in this study, clustered within genotype B1. Notably, our strains showed close genetic relationships with contemporaneous isolates from Thailand (2020), Yunnan, China (2023), and Shandong, China (2024), forming the B1b subcluster. In contrast, genotype B2 exclusively comprised historical strains from Japan and Malaysia isolated prior to 2000. The bootstrap values for clusters B1A, B1B, B1, and B2 were relatively low (ranging from 31 to 49), suggesting limited resolution at these nodes. Nevertheless, the sequences of interest formed a well-supported and distinct cluster within B1b.

EV-B88 was detected in sewage samples during February to April 2024, yielding partial VP1 sequences (378 bp, spanning nt 2,525–2,902 relative to prototype strain AY843306/BAN01-10398/Bangladesh/2007). As of June 5, 2025, only 16 EV-B88 nucleotide sequences were available in GenBank, none of which originated from China. Pairwise alignment revealed 78.6%–96.2% nucleotide similarity between our sequences and global references. Phylogenetic analysis delineated global EV-B88 strains into three well-supported clusters (A–C) (Fig. 2B). The prototype strain segregated within cluster A, whereas all sequences from this study formed a distinct subclade within cluster B. Notably, these strains exhibited the closest genetic relationship with environmental isolates from Pakistan (2013–2015), suggesting a potential regional circulation pattern.

EV-C99 was detected in two environmental samples collected in March and April 2024, yielding partial VP1 sequences of 291 bp, spanning nt 2,637–2,927 of prototype strain EF015008/BAN00-10461/2000. Comparative analysis included 29 GenBank-derived sequences (>300 bp), five of which originated from China. The nucleotide similarity between our sequences and the prototype strain ranged from 77.0% to 85.6%. Phylogenetic reconstruction revealed that the newly identified EV-C99 strains formed a distinct lineage, exhibiting substantial genetic divergence from both domestic and international references (Fig. 2C). Specifically, they clustered distantly from earlier Chinese isolates (Shandong, 2011; Yunnan, 2013) and contemporary foreign strains, including Italian wastewater isolates (2022–2023) and a Russian clinical strain (2023). This suggests potential undetected circulation or unique evolutionary dynamics.

A partial VP1 sequence of EV-D68 (373 bp, nt 2,487–2,859 relative to prototype strain KU844179/CA/US/Fermon/1962) was obtained from sewage in July 2024. Phylogenetic comparison with 38 global reference sequences (2007–2024) revealed nucleotide similarities ranging from 82.3% to 99.7%. Contemporary strains showed significant divergence from the historical Fermon prototype. The study sequence clustered within subclade A2, demonstrating close genetic relationships with 2024 isolates from the US, Italy, and China—particularly with a virulent strain from Shanghai, China (Fig. 2D). Phylogenetic reconstruction revealed dynamic genotypic shifts, with pre-2010 strains dominated by genotype C, followed by the emergence of subclade A1 (2012–2013), genotype B expansion (2013–2019), and contemporary A2 predominance (2019–2024) (Fig. 2D).

## DISCUSSION

This study presents a comprehensive analysis of EV surveillance in Jinan City’s sewage system during 2024, employing both cell culture–virus isolation and NGS technologies to compare their efficacy in EV detection. Our findings reveal significant differences in serotype identification between these methods, provide detailed characterization of circulating serotypes, with particular focus on epidemiologically important EVs and newly emerging variants, offering valuable insights into EV epidemiology and evolution.

Next-generation sequencing technology offers significant advantages for EV surveillance by enabling direct viral RNA extraction from concentrated sewage samples, thereby circumventing the serotype bias inherent in cell culture-based methods. Despite its demonstrated utility, the application of NGS for EV environmental surveillance remains limited in China except for pioneer studies in Shandong province since 2018 (6, 11, 18), which employed NGS on a 380-nt segment of the EV VP1 region or a 4 kb fragment encompassing the complete P1 coding region. Notably, during the COVID-19 pandemic (2019–2021), NGS-based environmental surveillance in Shandong Province identified 33 EV types, with E11, CVA10, E18, CVB4, and CVB5 emerging as the most prevalent, and including newer types EVA76, A89, A90, and C113, underscoring NGS’s superior capability in comprehensive EV surveillance (11). The current 2024 surveillance data from Jinan City reveal a distinct shift in EV type prevalence, with CVB4, CVA16, and E11 now dominating, contrasting sharply with the earlier predominance of E11, CVA10, and E14 during the pandemic period. Specifically, we observed a marked increase in CVB4 and CVA16 detection frequencies, accompanied by decreased prevalence of CVA10 and E14. These findings suggest cyclical patterns in EV circulation within the region, providing critical insights for understanding EV epidemiology and informing public health intervention strategies. The observed serotype fluctuations highlight the importance of sustained environmental surveillance to track EV evolutionary dynamics and implement early warning of potential outbreaks.

CVA16 was the predominant EV serotype identified in this study. It is a significant pathogen causing hand-foot-and-mouth disease (HFMD) in children worldwide, frequently co-circulating with EV-A71. Notably, since the widespread implementation of EV-A71 vaccines, the prevalence of CVA16 has gradually increased and shows a trend of surpassing that of EV-A71 (19). Global CVA16 strains can be divided into four major genogroups (A–D). Genogroup A contains the South African prototype strain. Genotypes C (classified by VP4 sequence) and D remain relatively uncommon. The predominant genotype B has evolved into three distinct sub-lineages (B1–B3), with B1 further differentiating into two common subgenotypes: B1a and B1b. Currently, B1b is the predominant global genotype, with widespread circulation across East Asia, Southeast Asia, Europe, and North America (20). Of particular concern was the 2019 CVA16 outbreak in Yantai, Shandong Province, which was attributed to the introduction of B1a strains from Southeast Asia (21). Phylogenetic analysis reveals that the 2024 Shandong strains cluster closely with both the 2020 Thailand isolates and 2023 Yunnan strains within the B1b subgenotype. These findings underscore the persistent dominance of B1b in current CVA16 transmission and the ongoing risk of cross-border viral spread. Enhanced surveillance systems and preparedness measures are urgently needed to monitor and respond to potential CVA16 outbreaks resulting from international viral transmission.

EV-B88 was detected with relatively high frequency (5/12) in this study, although it remains an uncommon type given the limited number of reported cases in the literature and the scarcity of available nucleotide sequences (*n* = 16) in GenBank. The prototype strain (AY843306) was initially isolated from the stool sample of a child with AFP in Bangladesh in 2007 (22), with subsequent detections reported in fecal samples from AFP cases in India between 2007 and 2009 (23) and in Senegal in 2017 (24). EV-B88 was also identified in acute gastroenteritis children in Thailand from 2010 to 2014 (25). Currently, all EV-B88 sequences deposited in GenBank originate from Bangladesh, India, Pakistan, Senegal, Russia, and Thailand, spanning from 2007 to 2023. This study documents the first identification of EV-B88 in China. The observed genetic divergence exceeding 3.8% between our sequences and those available in GenBank implies that EV-B88 was not recently introduced into the local region but has likely been circulating for a considerable period.

Since its initial detection in 2000, EV-C99 has been identified in numerous countries, including Bangladesh, the United States, Nigeria, the Philippines, China, Malawi, Italy, Thailand, and Russia, with growing evidence suggesting its potential association with acute gastroenteritis (26) and AFP (27). In China, only seven EV-C99 sequences have been documented to date, comprising three strains from Xinjiang Province in 2011 (28), two strains from Xinjiang students in Shandong Province in the same year (29), and two additional strains from Yunnan Province in 2013 and 2016, respectively, with both Xinjiang and Yunnan being located in western China. Our detection of EV-C99 in sewage samples demonstrates its circulation in eastern China, indicating the establishment of local transmission in this region. Phylogenetic analysis revealed significant genetic divergence among Chinese EV-C99 strains, which segregated into multiple distinct clades (Fig. 2C), strongly suggesting the co-circulation of diverse lineages within mainland China, likely resulting from either multiple independent introductions or prolonged undetected transmission of the virus.

We detected one EV-D68 sequence in sewage collected in July 2024. First identified in the United States in 1962 (30), EV-D68 has gained global attention due to increasing reports of associated acute flaccid myelitis. China documented its first EV-D68 infection in 2016 and reported the first AFP-afflicted child in 2018 (B3 lineage) (31). This is the first report of EV-D68 in sewage. Notably, EV-D68 transmission declined during 2020–2021 under strict NPIs but resurged post-relaxation (32), triggering outbreaks among children in the United States (2022) and Brazil (2023) (33, 34). A 2024 Italian outbreak revealed both the prevalent B3 subgenotype and the re-emergent A2 subgenotype—the latter exhibiting unusual infectivity in elderly populations (35). Phylogenetic analysis showed our sequence clusters closely with the 2024 Italian A2 strain and a contemporaneous pathogenic strain from Shanghai, suggesting a potential local spread of this emerging lineage.

This study has several limitations. Compared with the cell culture method, NGS failed to detect serotype E7, despite successfully identifying all eight other EV serotypes. Also, a pronounced contrast was noted for CVB4 (2/12 by culture vs. 9/12 by NGS) and E3 (8/12 vs. 2/12). Whether this divergence stems from random sampling error due to the limited sample size or reflects a systematic methodological bias requires further study. Furthermore, the NGS approach generated partial VP1 sequences (~350 nt), which may not fully capture the genetic diversity represented by the complete VP1 region. Future studies should employ more sensitive and efficient amplification methods to obtain the entire VP1 coding sequence, ensuring more comprehensive genomic characterization.

### Conclusions

In this study, we conducted environmental surveillance of EVs in Jinan City by employing both cell culture and NGS technologies in parallel. Our analysis revealed the genetic diversity of EVs and RVs in sewage samples, contributing to a deeper understanding of the molecular epidemiology of emerging EV strains. Furthermore, this work highlights the utility of NGS in monitoring regional EV circulation, providing valuable insights for public health surveillance.

## Data Availability

The sequences have been deposited in the GenBank database under accession numbers PV747863–PV747892.
